# Effects of Water Acidification on Senegalese Sole *Solea senegalensis* Health Status and Metabolic Rate: Implications for Immune Responses and Energy Use

**DOI:** 10.3389/fphys.2020.00026

**Published:** 2020-01-31

**Authors:** Marina Machado, Francisco Arenas, Jon C. Svendsen, Rita Azeredo, Louis J. Pfeifer, Jonathan M. Wilson, Benjamín Costas

**Affiliations:** ^1^Centro Interdisciplinar de Investigação Marinha e Ambiental, Universidade do Porto, Matosinhos, Portugal; ^2^Instituto de Ciências Biomédicas Abel Salazar, Universidade do Porto, Porto, Portugal; ^3^National Institute of Aquatic Resources, Technical University of Denmark, Charlottenlund, Denmark; ^4^Biology Department, Wilfrid Laurier University, Waterloo, ON, Canada

**Keywords:** water acidification, flatfish, gills immunofluorescence, respirometry, immune system

## Abstract

Increasing water CO_2_, aquatic hypercapnia, leads to higher physiological pCO_2_ levels in fish, resulting in an acidosis and compensatory acid-base regulatory response. Senegalese sole is currently farmed in super-intensive recirculating water systems where significant accumulation of CO_2_ in the water may occur. Moreover, anthropogenic releases of CO_2_ into the atmosphere are linked to ocean acidification. The present study was designed to assess the effects of acute (4 and 24 h) and prolonged exposure (4 weeks) to CO_2_ driven acidification (i.e., pH 7.9, 7.6, and 7.3) from normocapnic seawater (pH 8.1) on the innate immune status, gill acid-base ion transporter expression and metabolic rate of juvenile Senegalese sole. The acute exposure to severe hypercapnia clearly affected gill physiology as observed by an increase of NHE3b positive ionocytes and a decrease of cell shape factor. Nonetheless only small physiological adjustments were observed at the systemic level with (1) a modulation of both plasma and skin humoral parameters and (2) an increased expression of HIF-1 expression pointing to an adjustment to the acidic environment even after a short period (i.e., hours). On the other hand, upon prolonged exposure, the expression of several pro-inflammatory and stress related genes was amplified and gill cell shape factor was aggravated with the continued increase of NHE3b positive ionocytes, ultimately impacting fish growth. While these findings indicate limited effects on energy use, deteriorating immune system conditions suggest that Senegalese sole is vulnerable to changes in CO_2_ and may be affected in aquaculture where a pH drop is more prominent. Further studies are required to investigate how larval and adult Senegalese sole are affected by changes in CO_2_.

## Introduction

Senegalese sole (*Solea senegalensis*, Kaup) is currently reared in super-intensive recirculation aquaculture systems (RAS) (Morais et al., 2016). This production structure allows high rearing densities together with a minimal water change (Martins et al., 2009). Such efficiency depends on the constant control and maintenance of water parameters, guaranteeing optimal fish performance (Bowden, 2008). Still, because of CO_2_ accumulation in the water, there is a possible decrease of pH (Summerfelt et al., 2000) throughout the system, ultimately creating areas where acute or prolonged hypercapnia may be found. In fact, in salmonids production the concentration of CO_2_ can reach 10–20 mg L^–1^ (Mota et al., 2019), while the guidelines for intensive RAS production has proposed a safe limit of 40 mg L^–1^ CO_2_ (Blancheton, 2000; Ellis et al., 2017). Moreover, as a known consequence of anthropogenic activity, the release of CO_2_ into the atmosphere is absorbed by the oceans leading to the decrease of oceanic water pH (Bresolin de Souza et al., 2014). In both scenarios, and despite the great despair in pH values found in aquaculture and in oceans (Ellis et al., 2017), the increase of water CO_2_ leads to higher physiological pCO_2_ in the fish. This could impact fish acid-base balance, which results in the activation of regulatory mechanisms to compensate for the acidosis (Melzner et al., 2009) and initiates reflexes such as bradycardia and hyperventilation (Perry and Abdallah, 2012). Perry and Abdallah (2012) reviewed that changes in water CO_2_ can be sensed by chemoreceptors mainly located in fish gills that are involved in elevating plasma HCO_3_^–^ concentrations to compensate for the respiratory acidosis (Heuer and Grosell, 2014; Allmon and Esbaugh, 2017; Esbaugh, 2018). The marked effects of modest elevations in CO_2_ in fish are due in part to the low physiological pCO_2_ levels found in fish (Qin et al., 2010; Clements and Chopin, 2017). The maleficent effects of aquatic hypercapnia rest on CO_2_ concentration and duration of the exposure (Santos et al., 2013), and besides being a stress factor, hypercapnia can also be responsible for energy deviation from growth and other metabolic processes (Cech and Crocker, 2002; Stapp et al., 2015). Elevated water CO_2_ concentration is associated with a drop of extra- and intra-cellular pH and a concomitant depression of cytosolic and membrane enzymes activity, ion transport and protein and DNA biosynthesis (Busa and Nuccitelli, 1984). In fact, juvenile spotted wolfish (*Anarhichas minor* Olafsen) showed reduced growth and plasma chloride levels after 10 weeks exposure to pH 6.45 (Foss et al., 2003) while Atlantic salmon post-smolt (*Salmo salar*) growth decreased linearly with the increase of water CO_2_ (Mota et al., 2019). Moreover, Bresolin de Souza et al. (2014) observed an up-regulated expression of plasma immune system- and gill metabolism- related proteins in response to high CO_2_. In addition, Wang et al. (2010) showed that elevated CO_2_ inhibits LPS-induced expression of IL6 and TNF and impairs bacterial clearance through inhibition of both phagocytosis and autophagy in macrophages. Atlantic halibut (*Hippoglossus hippoglossus*) exposed to high CO_2_ showed up-regulation of plasma immune-related proteins as the complement component C3 and gills metabolism- and cellular turnover- related proteins, possibly due to an increase of energy demand (Bresolin de Souza et al., 2014). Hence, the fish immune system could also be affected acutely by hypercapnia since immune cells internal milieu is directly affected and reflects the external environment (Boleza et al., 2001).

Short-term hypercapnia exposure is generally alleviated through small adjustments in fish metabolism (Cech and Crocker, 2002), but in response to a chronic exposure or to increased ambient CO_2_ level, the metabolic demands may increase and reach the limit of the regulatory mechanisms. In fact, it is suggested that as soon as the compensatory mechanisms are exceeded, fish adopt a passive strategy toward the stress resolution by shifting the available energy to survival related machinery (Walsh et al., 1988; Pörtner et al., 1998; Baker and Brauner, 2012; Stapp et al., 2015). Few studies have investigated the effect of hypercapnia on fish immune mechanisms, which is particularly true for flatfishes. Since fish immunity is highly dependent on both host energy budget and environmental variables, this study assessed the effects of acute and prolonged exposures to CO_2_ induced low pH levels on the innate immune status, gill acid-base ion transport and metabolic rates of Senegalese sole. This was accomplished using juvenile fish exposed to pH 7.9, 7.6, and 7.3 and sampled after 4, 24 h and 4 weeks of exposure.

## Materials and Methods

### Experimental Design

The trial was performed at the Centro Interdisciplinar de Investigação Marinha e Ambiental (CIIMAR) facilities in Porto, Portugal. Senegalese sole juveniles (82.74 ± 0.75 g) were obtained from a commercial fish farm, located in north-west Portugal (Aquacria, Aveiro, Portugal). Fish were acclimated for one month in a recirculating sea water system according to standard protocols, with continuous control of water parameters and were daily fed a commercial diet (2% body weight). Water was kept at 19°C, salinity at 24 ppt, dissolved oxygen at 100% air saturation (O_2__sat_), and a photoperiod of 12 h light/12 h dark was adopted. After being weighed, 24 fish were distributed into four independent recirculating seawater systems (*n* = 6 per tank) comprised by a header tank and 6 flat-bottomed tanks (0.05 m^2^; Temp.: 19 ± 1°C; Salinity: 24 ppt; O_2__sat_: 100%; pH = 8.1, 0.756 mg l^–1^ CO_2_). Each of the systems comprised a biological and mechanical biofiltration system. The water pH of each system was controlled by a pH-stat system (Aqua Medic^®^, AT Control-SW, version 9.0) (Wilcox-Freeburg et al., 2013). A solenoid valve was used to regulate the delivery of compressed CO_2_ gas into the water of the respective header tanks to establish and maintain experimental pH levels at 7.9, 7.6, or 7.3 with an expected final concentration of 1.253, 2.601, and 5.297 mg l^–1^ of CO_2_, respectively, and calculated according to Mojica Prieto and Millero (2002). The solenoid valves were activated when pH values were 0.1 pH units above their pre-set levels. The pH values of all header tanks were recorded with a data-logger. Control conditions were secured in a fourth system with normal seawater pH (8.1). One fish per tank (*n* = 6) was sampled 4 and 24 h after the beginning of the trial to assess the acute effects of a decrease in pH. The remaining fish were fed with a commercial diet (2.5% biomass per day) and were weighed and sampled after a 4-week period for the assessment of the prolonged water acidification. At all sampling times, fish were fasted for 24 h prior to sampling, anesthetized by immersion in 2-phenoxyethanol (1500 ppm; Sigma-Aldrich, St. Louis MO, United States) and sampled for skin mucus, blood, gills and head-kidney tissue (HK). A second trial was performed, with the identical water conditions, fish size and pH treatments. A final number of 12 fish per treatment was used for analysis of hematology, plasma and mucus humoral parameters.

For respirometry assays, six fish were maintained in an ambient tank at the desired pH (i.e., pH levels at 8.1, 7.9, 7.6, or 7.3) for 1 week acclimatization (Temp.: 19 ± 1°C; Salinity: 24 ppt; O_2_ saturation: 100%) and fasted for 24 h before being chased to exhaustion and placed in the respirometer chamber for 24 h. Following previous studies (Svendsen et al., 2012; Baktoft et al., 2016; Peixoto et al., 2016), chasing procedures were used to induce the maximum metabolic rate (MMR), whereas the standard metabolic rate (SMR) was estimated using respirometry data collected over the subsequent 24 h. Aerobic metabolic scope (AMS) was estimated by subtracting SMR from the MMR (Svendsen et al., 2014).

The experiments were approved by the Animal Welfare Committee of the Interdisciplinary Centre of Marine and Environmental Research and carried out in a registered installation (N16091.UDER). Trained scientists performed experiments in full compliance with national rules and following the European Directive 2010/63/EU of the European Parliament and the European Union Council on the protection of animals used for scientific purposes.

### Fish Growth Performance

All fish were weighted at the beginning of the trial and 12 fish per treatment (*n* = 1 per tank) were weighted at 4 weeks of exposure to the different pH. Weight gain (WG) and specific growth rate (SGR) were calculated as follows:

$$\mathrm{WG}=\frac{\mathrm{Final}\,\mathrm{weight}\times 100}{\mathrm{Inital}\,\mathrm{weight}}-100$$

$$\mathrm{SGR}=\frac{\left[\ln\left(\mathrm{Final}\,\mathrm{weight}\right)-\text{ln}\,\left(\mathrm{Initital}\,\mathrm{weight}\right)\right]\times 100}{\mathrm{time}\,\left(\mathrm{days}\right)}$$

### Tissue Collection

Skin mucus was gently collected from the skin surface of each fish using a cell scraper and transferred into 2 ml Eppendorf tube, and centrifuged at 400 × *g* for10 min at 4°C to remove possible cells and scales. Blood was collected from the caudal vessels using heparinized syringes. A subsample was used for hematological analysis and the remainder centrifuged at 10,000 × *g* for 10 min at 4°C. After centrifugation, plasma was collected and immediately stored at −80°C for evaluation of innate immune parameters. Head-kidney tissues were excised from all fish and immediately stored at −80°C. The second gill arch of each fish was excised and immersion-fixed in 10% neutral buffered formalin (pH 7.4) for 24 h at 4°C and stored in 70% ethanol for later paraffin embedding.

### Analysis of Hematological Parameters

The hematological profiling was conducted according to Machado et al. (2015) and consisted of measurements of hematocrit (Ht), hemoglobin (Hb; SPINREACT kit, ref. 1001230, Spain), and total white (WBC) and red (RBC) blood cells counts. Subsequently, the mean corpuscular volume (MCV), mean corpuscular hemoglobin (MCH), and mean corpuscular hemoglobin concentration (MCHC) were calculated. Also, blood smears were made, air dried, and after fixation as described by Machado et al. (2015), the absolute value (× 10^4^ ml^–1^) of each cell type (neutrophils, monocytes, lymphocytes and thrombocytes) was calculated based on total blood WBC count.

### Plasma and Skin Mucus Innate Humoral Parameters

#### Plasma Lysozyme

Lysozyme activity was estimated in plasma samples and measured using a turbidimetric assay as described by Costas et al. (2011). Briefly, a solution of *Micrococcus lysodeikticus* (0.5 mg ml^–1^, 0.05 M sodium phosphate buffer, pH 6.2) was prepared. To a microplate, 15 μl of plasma in triplicates and 250 μl of the above suspension were added to give a final volume of 265 μl. The reaction was carried out at 25°C and the absorbance (450 nm) was measured after 0.5 and 4.5 min in a Synergy HT microplate reader (BioTeck Instruments, Synergy HT). Lyophilized hen egg white lysozyme (Sigma) was serially diluted in sodium phosphate buffer (0.05 M, pH 6.2) and used to develop a standard curve. The amount of lysozyme in the sample was calculated using the formula of the standard curve.

#### Plasma and Mucus Peroxidase

Total peroxidase activity in plasma and skin mucus was measured following the procedure described by Quade and Roth (1997). Briefly, 15 μl of plasma or mucus in triplicates were diluted with 135 μl of Hank’s Balanced Salt Solution (HBSS) without Ca^+2^ and Mg^+2^ in flat-bottomed 96-well plates. Then, 50 μl of 20 mM 3,3′,5,5′-tetramethylbenzidine hydrochloride (TMB; Sigma) and 50 μl of 5 mM H_2_O_2_ were added. The color-change reaction was stopped after 2 min by adding 50 μl of 2 M sulfuric acid and the optical density was read at 450 nm in a Synergy HT microplate reader. Wells without plasma or mucus were used as blanks. The peroxidase activity (units ml^–1^ plasma or mg per protein mucus) was determined by defining one unit of peroxidase as that which produces an absorbance change of 1 OD. Mucus results were presented as units of peroxidase per milligram of protein.

#### Plasma and Mucus Total Protein and Plasma Immunoglobulin

Total protein concentrations were determined in 1:50 (v/v) diluted plasma and mucus samples using the bicinchoninic acid (BCA) Protein Assay Kit (Pierce #23225, Rockford, IL, United States) for microplates. Bovine serum albumin served as a standard. For immunoglobulins determination, the immunoglobulins were precipitated with polyethylene glycol in 1:2 (v/v) diluted plasma and mucus. After a 2 h incubation, samples were centrifuged for 10 min at 4472 × *g* and 10 μl of the supernatant was then diluted 1:50 (v/v) in distilled water and protein determined using the previous Protein Assay Kit. Total plasma and mucus immunoglobulins were determined by subtracting the values that result from the immunoglobulin precipitation by polyethylene glycol to the values obtained for total protein of the samples (Siwicki and Anderson, 1993).

#### Plasma and Mucus Antiprotease Activity

The anti-protease activity was determined as described by Ellis (1990) with modifications (Machado et al., 2015). Briefly, 10 μl of plasma or 40 μl mucus was incubated with 10 μl of a trypsin solution (5 mg ml^–1^ in 0.5% NaHCO_3_, pH 8.3) for 10 min at 22°C in polystyrene microtubes. To the incubation mixture, 100 μl of phosphate buffer (NaH_2_PO_4_, 13.9 mg ml^–1^, pH 7.0) and 125 ml of azocasein (20 mg ml^–1^ in 0.5% NaHCO_3_, pH 8.3) were added and incubated for 1 h at 22°C. Finally, 250 μl of 10% trichloroacetic acid (TCA) were added to each microtube and incubated for 30 min at 22°C. The mixture was centrifuged at 10,000 × *g* for 5 min at room temperature. Afterward, 100 μl of the supernatant was transferred to a 96 well-plate containing 100 μl of 1N NaOH per well. The OD was read at 450 nm in a Synergy HT microplate reader. Phosphate buffer in place of plasma and mucus and trypsin served as blank, whereas the reference sample was phosphate buffer in place of plasma and mucus. The inhibition percentage of trypsin activity was calculated based on the reference sample.

#### Plasma and Mucus Proteases Activity

For protease activity evaluation, 100 μl of 0.5% NaHCO_3_ (pH 8.3) and 125 μl azocasein (20 mg ml^–1^ in 0.5% NaHCO_3_, pH 8.3) was added to 10 μl of plasma or 100 μl of mucus. After incubation during 24 h at 22°C in polystyrene microtubes, 250 μl of 10% TCA was added and centrifuged (10,000 × *g* for 5 min) and the absorbance of 100 μl of the supernatant, plus 100 μl of 1N NaOH read at 450 nm in a Synergy HT microplate reader. The percentage protease activity compared to the reference sample (trypsin solution, 5 mg ml^–1^ in 0.5% NaHCO_3_, pH 8.3) was calculated (Ellis, 1990).

#### Plasma and Mucus Bactericidal Activity

*Photobacterium damselae* subsp. *piscicida, Phdp*, strain PP3, isolated from yellowtail (*Seriola quinqueradiata*; Japan) by Dr. Andrew C. Barnes (Marine Laboratory, Aberdeen, United Kingdom) was used in the bactericidal activity assay. Bacteria were cultured and exponentially growing bacteria were re-suspended in sterile HBSS and adjusted to 1 × 10^6^ CFU ml^–1^. Plating serial dilutions of the suspensions onto TSA-1 plates and counting the number of CFU following incubation at 22°C confirmed the bacterial concentration of the inoculum. Plasma bactericidal activity was then determined following the method described by Graham and Secombes (1988) with modifications (Machado et al., 2015). Briefly, 20 μl of plasma or mucus were added to duplicate wells of a U-shaped 96-well plate. HBSS was added to some wells instead of plasma and served as positive control. To each well, 20 μl of *Phdp* (1 × 10^6^ CFU ml^–1^) was added and the plate was incubated for 2.5 h at 25°C. Twenty-five μl of 3-(4, 5 dimethyl-2-yl)-2, 5-diphenyl tetrazolium bromide (1 mg ml^–1^; Sigma) was added to each well and incubated for 10 min at 25°C to allow the formation of formazan. Plates were then centrifuged at 2000 × *g* for 10 min and the precipitate was dissolved in 200 μl of dimethyl sulfoxide (Sigma). The absorbance of the dissolved formazan was measured at 560 nm. Bactericidal activity was expressed as a percentage, calculated from the difference between bacteria surviving compared to the number of bacteria from positive controls (100%). All analyses were conducted in triplicates.

### Gene Expression Analysis

Total RNA isolation was conducted with TRIzol Reagent (Invitrogen) following the manufacturer’s specifications. RNA was treated with DNase I (GRiSP Research Solutions, Porto, Portugal) to remove genomic DNA, and first-strand cDNA was synthesized with NZY First-Strand cDNA Synthesis Kit (NZYTech, Lisbon, Portugal). Quantitative PCR assays were performed with an iQ5 Real Time PCR detection System (Bio-Rad) using 1 μl of diluted cDNA (1:5 dilution) mixed with 10 μl of iQ SYBR green 2x Supermix (Bio-Rad) and 0.4 μl (10 mM) of each specific primer in a final volume of 20 μl. The cDNA amplifications were carried out with specific primer pairs (Table 1) for genes of interest, which were designed using NCBI Primer Blast Tool according to known qPCR restrictions (amplicon size, Tm difference between primers, GC content and self-dimer or cross-dimer formation). The efficiencies of the primer pairs were analyzed in serial five-fold dilutions of cDNA by calculating the slope of the regression line of the cycle thresholds (Ct) versus the relative concentration of cDNA (Livak and Schmittgen, 2001).

**TABLE 1:** Genes, real-time PCR primer information including gene names, symbols, reaction efficiencies, predicted product sizes, and primer sequences (5′-3′).

Gene name	Symbol	Gene bank	Eff ^a^	Product length^b^	Forward primer	Reverse primer
18S ribosomal	18S	EF126042.1	2.15	129	GGTCCGAAGCGTTTACTTTG	GCCCCAGTTCAGAGAGAGAA
Interleukin 1 β	IL1-β	KU695545	2.06	127	ACTGAGGCCAGACCTGTAGC	TGACGGTTGACAGACAGCTT
Ciclo-oxigenase-2^c^	COX 2	-	2.07	100	CCGACTTACAATGCGGATTATGGTT	TTGGGCAATCCTCTGGTACAG
Glucocorticoid receptor 1	GR1	AB614369.1	2.11	154	CATGACGACCCTGAACCGAT	CCAGCCCAGACTGAAAGACA
Interleukin 10^c^	IL10	-	2.06	78	CCGTCTTTGTGTTATTTCTCCAACAG	TGGAGTTCAGCTTTGTGATGTCA
Hypoxia Inducible factor 1	HIF-1	FF288855.1	1.96	140	TGCCACGCAGTAAAAATCAG	CTTCACCGCTTTGACTGTA

*^*a*^Efficiency of PCR reactions were calculated from serial dilutions of tissue RT reactions in the validation procedure. ^*b*^Amplicon (nt). ^*c*^Transcript sequences were retrieved from SoleaDB (http://www.juntadeandalucia.es/agriculturaypesca/ifapa/soleadb_ifapa/).*

Efficiency values, complete sequences and product length are presented in Table 1. Melting curve analysis was also performed to verify that no primer dimers were amplified. The standard cycling conditions were 95°C for 10 min, followed by 40 cycles of 95°C for 15 s and 60°C for 1 min. All reactions were carried out as technical duplicates. The Ct values were compared through the comparative Ct method to the geometric mean derived from the expression of Senegalese sole 18S ribosomal (18S) (Livak and Schmittgen, 2001). Fold change units were calculated by dividing the normalized expression values of tissue from different treatments by the normalized expression values of normocapnia at 4 h treatment group.

### Gills Immunofluorescence

The formalin fixed gill tissue was decalcified and processed for paraffin embedding. Gills were sectioned (5 μm) using a Leica RM2235 RTS microtome. The sections were collected on APS coated glass slides, air-dried, de-waxed and rehydrated. Following a blocking step with BLØK blocking buffer (Merck KGaA, Darmstadt, Germany), immunohistochemical staining was accomplished using primary antibodies against key ion-acid-base transporters, which included antibodies against the- Na^+^/K^+^ ATPase α subunit, Na^+^: K^+^: 2Cl^–^ cotransporter (NKCC), cystic fibrosis transmembrane regulator (CFTR), Na^+^/H^+^ exchanger 3b (NHE3b), cytosolic carbonic anhydrase I and II (CA I and CA II, respectively). See Table 2 for optimal dilutions, antigen retrieval and antibody incubation conditions. All antibodies were diluted in BLØK and further information on these antibodies can be found in Table 2. The following antibody pairs were used for double immunolabeling of the gill tissue sections as outlined in Table 3. Secondary antibodies, which included goat anti-mouse (Alexa555) and goat anti-rabbit (Alexa488), were diluted 1:500 in BLØK and incubated with sections for 1 h at 37°C. Sections were rinse in TPBS (0.05% Tween-20 in Phosphate Buffered Saline, pH 7.4) and counterstained with DAPI (4′,6-Diamidine-2′-phenylindole dihydrochloride) and viewed with a Leica DM5500B photomicroscope using Leica LASX software and a Hamamatsu Orca Flash 4 camera. Images immunolabelled for NKA (α5) were analyzed using SigmaScan Pro software for cell average size (area), average (green or red) fluorescence area-intensity and shape-factor. Gill ionocyte counts were also made and expressed as either per length of the gill filament or per interlamellar space. The proportion of NHE3b positive NKA-IR cell was also made.

**TABLE 2:** Antibodies used for immunofluorescence microscopy.

Antibody	Origin
Na^+^/K^+^-ATPase α subunit (Rabbit Polyclonal αR1)	Wilson et al., 2007
NKCC (Mouse Monoclonal; T4 clone)	Developmental Studies Hybridoma Bank (Iowa, United States)
CFTR (cystic fibrosis transmembrane conductance factor)	R&D Systems
Na^+^/K^+^ ATPase α subunit (Mouse Monoclonal; α5 clone)	Developmental Studies Hybridoma Bank (Iowa, United States)
NHE3b (Na^+^/H^+^ exchanger)	Hirose
CA I (Carbonic Anhydrase I)	Abcam
CA II (Carbonic Anhydrase II)	Abcam
Alexa Fluor 488 (Goat anti rabbit)	Invitrogen (Carlsbad, CA, United States)
Alexa Fluor 568 (Goat anti mouse)	Invitrogen (Carlsbad, CA, United States)

**TABLE 3:** Antibody combinations and dilutions used for immunofluorescence microscopy.

Combination	Primary	Dilution	Secondary	Dilution
	antibodies		antibodies	
NHE3b and α5	NHE3b	1:1000	Alexa 488	1:500
	α5	1:100	Alexa 568	1:500
NHE3b and NKCC	NHE3b	1:1000	Alexa 488	1:500
	NKCC	1:100	Alexa 568	1:500
αR1 and α5	αR1	1:500	Alexa 488	1:500
	α5	1:100	Alexa 568	1:500
αR1 and CFTR	αR1	1:500	Alexa 488	1:500
	CFTR	1:50	Alexa 568	1:500
CAI and α5	CAI	1:100	Alexa 488	1:500
	α5	1:100	Alexa 568	1:500
CAII and α5	CAII	1:100	Alexa 488	1:500
	α5	1:100	Alexa 568	1:500

### Respirometry

Metabolic rates were measured by intermittent flow respirometry as described by Svendsen et al. (2012). Static respirometer chambers (0.6 L) were used to measure oxygen consumption rate (ṀO_2_; mg O_2_ kg^–1^ h^–1^) at the different pH values. Six fish were maintained in an ambient tank at the desired pH (pH 8.1, 7.9, 7.6, or 7.3) for 1 week acclimatization (Temp.: 19 ± 1°C; Salinity: 24 ppt; O_2_ air saturation: 100%) and fasted for 24 h before being placed in the respirometer chamber. Oxygen partial pressure (kPa) inside the respirometers was measured using galvanic sensor technology (Mini DO Probe; Loligo Systems; Denmark). The software AutoResp (Loligo Systems Aps, Tjele, Denmark) collected the data every 9 min, and calculated ṀO_2_ from measurements of oxygen content inside the respirometers, adjusted for the body mass of the individual fish. The respirometer was submerged in the ambient tank supplying water at the desired pH (pH 8.1, 7.9, 7.6, or 7.3) for the respirometer. A standard protocol was used to induce MMR where individual fish were handled for 3 min and then exposed to 2 min of air exposure. Following exhaustion, fish were transferred to the respirometers where ṀO_2_ recordings started immediately. MMR was estimated as the average of the highest three consecutive measures of ṀO_2_ recorded immediately after placement of the fish inside the respirometry chamber. Next, fish were left undisturbed in the respirometers for 24 h and ṀO_2_ values were logged continuously. SMR was estimated as the average of the 10% lowest MO_2_ values (Baktoft et al., 2016; Peixoto et al., 2016). AMS was calculated as the difference between MMR and SMR (Svendsen et al., 2014).

### Statistical Analysis

All results are expressed as mean ± standard deviation (mean ± SD). Data were analyzed for normality and homogeneity of variance and, when necessary, transformed before being treated statistically. All data expressed as percentage were arcsine transformed (Zar, 1999). Data were analyzed by two-way ANOVA, with time and pH as factors, followed by the Tukey *post hoc* test to identify differences in the experimental treatments. All statistical analyses were performed using the computer package STATISTICA 12 for WINDOWS. The level of significance used was *P* ≤ 0.05 for all statistical tests. Finally, one-way ANOVAs were used to examine the effects of pH on growth estimates and the metabolic rate data.

## Results

### Fish Growth Performance

After 4 weeks of exposure to the different pH, fish held at pH 7.3 displayed lower final body weight than those fish held at pH 8.1 and 7.9, indicating reduced growth among the fish exposed to elevated CO_2_ levels (Table 4).

**TABLE 4:** Data on the initial weight (g) and final weight (g), weight gain (%) and specific growth rate (%) of Senegalese sole exposed to different water pH levels over 4 weeks.

Parameters	pH				One-way ANOVA (*p*-value)
	8.1	7.9	7.6	7.3	
Initial weight	82.48 ± 0.16	83.125 ± 0.77	83.67 ± 0.11	81.67 ± 0.42	ns
Final weight	89.68 ± 15.05^a^	87.18 ± 17.28^a^	84.32 ± 9.48^ab^	76.37 ± 3.69^b^	0.043
Weight gain	8.73 ± 18.24	4.87 ± 11.45	0.77 ± 11.38	−6.49 ± 4.51	ns
Specific growth rate	0.32 ± 0.77	0.12 ± 0.97	0.01 ± 0.50	−0.31 ± 0.22	ns

*Values are presented as means ± SD (*n* = 6). *P*-values from one-way ANOVA (*p* ≤ 0.05). If interaction was significant, Tukey *post hoc* test was used to identify differences in the experimental treatments. Different lowercase letters indicate differences among pH.*

### Hematological and Peripheral Leukocytes Responses

Data regarding fish hematological profiles and differential peripheral leukocyte counts can be found in Tables 5, 6. Total WBC, lymphocyte and monocyte concentrations increased in fish exposed to pH 7.3 compared to those exposed to pH 7.6, 7.9, and 8.1 regardless time. Similarly, fish exposed to the lowest pH revealed a higher concentration of thrombocytes compared to those under pH treatments 7.9 and 8.1. Lymphocyte numbers also augmented in fish held at pH 7.6 relative to the control pH (8.1). Finally, neutrophil concentration increased in fish held at pH 7.3 compared to those exposed to pH 7.6 and 8.1. Regardless of pH treatment, Ht, MCV, total WBC, thrombocytes, lymphocytes and neutrophils values showed an increase in time.

**TABLE 5:** Hematocrit (%), hemoglobin (g dl), mean corpuscular volume (MCV; μm^3^), mean corpuscular hemoglobin (MCH; pg cell^–1^), mean corpuscular hemoglobin concentration (MCHC; g 100 ml^–1^), red blood cells [RBC; (× 10^6^ μl)], and white blood cells [WBC; (× 10^4^ μl)] of Senegalese sole submitted to different water pH during 4, 24 h and 4 weeks.

Parameters	pH											
	8.1			7.9			7.6			7.3		
	4 h	24 h	4 weeks	4 h	24 h	4 weeks	4 h	24 h	4 weeks	4 h	24 h	4 weeks
Hematocrit	8.27 ± 1.88	7.53 ± 0.93	9.17 ± 2.11	7.25 ± 1.35	8.17 ± 2.80	11.20 ± 2.23	7.72 ± 1.77	7.73 ± 1.16	9.00 ± 1.79	7.84 ± 1.46	9.01 ± 1.78	9.20 ± 2.23
Hemoglobin	1.78 ± 0.10	1.79 ± 0.10	1.79 ± 0.10	1.77 ± 0.14	1.81 ± 0.18	1.69 ± 0.11	1.80 ± 0.07	1.79 ± 0.17	1.69 ± 0.09	1.85 ± 0.15	1.88 ± 0.18	1.79 ± 0.18
MCV	74.72 ± 4.61	86.99 ± 21.17	126.17 ± 18.65	88.35 ± 35.12	91.94 ± 36.64	114.30 ± 5.62	94.06 ± 43.98	107.87 ± 42.28	126.05 ± 24.97	70.55 ± 55.76	101.57 ± 15.84	137.62 ± 31.42
MCH	17.02 ± 4.50	21.12 ± 6.24	25.68 ± 5.32	24.42 ± 10.67	20.43 ± 3.71	18.75 ± 4.22	21.63 ± 5,27	26.59 ± 6.22	23.56 ± 5.76	24.43 ± 4.94	21.13 ± 3.36	24.40 ± 5.92
MCHC	22.58 ± 4.93	24.14 ± 3.40	21.17 ± 7.20	25.16 ± 5.55	20.88 ± 3.48	15.68 ± 3.81	24.44 ± 4.96	23.37 ± 3.81	19.82 ± 5.17	24.65 ± 2.97	21.48 ± 3.33	20.00 ± 4.42
RBC	1.12 ± 0.29	0.92 ± 0.26	0.72 ± 0.11	0.82 ± 0.24	0.90 ± 0.11	0.94 ± 0.19	0.88 ± 0.21	0.72 ± 0.20	0.75 ± 0.14	0.78 ± 0.14	0.85 ± 0.29	0.76 ± 0.17
WBC	11.88 ± 1.15	12.75 ± 1.11	16.08 ± 2.52	13.68 ± 4.08	15.52 ± 3.68	19.18 ± 3.03	14.96 ± 3.22	16.19 ± 5.06	21.83 ± 2.63	16.49 ± 4,04	21.44 ± 7.38	27.37 ± 6.11

**Two-way ANOVA**						**pH**				**Time**		
**Parameters**	**pH**		**Time**	**pH × Time**		**8.1**	**7.9**	**7.6**	**7.3**	**4 h**	**24 h**	**4 weeks**

Hematocrit	ns		0.002	ns		–	–	–	–	B	B	A
Hemoglobin	ns		ns	ns		–	–	–	–	–	–	–
MCV	ns		<0.001	ns		–	–	–	–	A	A	B
MCH	ns		ns	ns		–	–	–	–	–	–	–
MCHC	ns		ns	ns		–	–	–	–	–	–	–
RBC	ns		ns	ns		–	–	–	–	–	–	–
WBC	<0.001		<0.001	ns		B	B	B	A	C	B	A

*Values are presented as means ± SD (*n* = 12). *P*-values from two-way ANOVA (*p* ≤ 0.05). If interaction was significant, Tukey *post hoc* test was used to identify differences in the experimental treatments. Different capital letters indicate differences among pH regardless time or among times regardless pH.*

**TABLE 6:** Absolute values (× 10^4^μl) of peripheral blood leukocytes (thrombocytes, lymphocytes, monocytes, and neutrophils) of Senegalese sole submitted to different water pH during 4, 24 h and 4 weeks.

Parameters	pH											
	8.1			7.9			7.6			7.3		
	4 h	24 h	4 weeks	4 h	24 h	4 weeks	4 h	24 h	4 weeks	4 h	24 h	4 weeks
Thrombocytes	7.14 ± 1.08	6.47 ± 1.23	9.32 ± 1.26	7.32 ± 1.86	7.33 ± 1.73	9.81 ± 1.22	7.65 ± 1.46	8.04 ± 2.05	11.15 ± 1.14	7.76 ± 1.20	9.09 ± 4.18	14.64 ± 3.96
Lymphocytes	4.31 ± 1.00	6.02 ± 1.24	5.28 ± 1.86	5.65 ± 2.58	6.99 ± 1.84	7.19 ± 3.16	6.49 ± 1.91	6.82 ± 2.17	9.69 ± 1.86	7.68 ± 3.18	10.06 ± 3.39	9.81 ± 3.04
Monocytes	0.14 ± 0.11	0.13 ± 0.14	0.23 ± 0.20	0.17 ± 0.14	0.36 ± 0.35	0.27 ± 0.21	0.21 ± 0.15	0.31 ± 0.41	0.17 ± 0.13	0.29 ± 0.15	0.41 ± 0.36	0.68 ± 0.65
Neutrophils	0.30 ± 0.17	0.13 ± 0.20	1.26 ± 0.56	0.53 ± 0.58	0.84 ± 0.99	1.95 ± 1.63	0.60 ± 0.52	0.92 ± 1.10	0.81 ± 0.37	0.75 ± 0.42	1.88 ± 0.73	2.24 ± 1.27

**Two-way ANOVA**						**pH**				**Time**		
**Parameters**	**pH**		**Time**	**pH × Time**		**8.1**	**7.9**	**7.6**	**7.3**	**4 h**	**24 h**	**4 weeks**

Thrombocytes	<0.001		<0.001	ns		B	B	AB	A	B	B	A
Lymphocytes	<0.001		0.008	ns		C	BC	B	A	A	B	B
Monocytes	0.008		ns	ns		B	B	B	A	–	–	–
Neutrophils	<0.001		<0.001	ns		B	AB	B	A	B	B	A

*Values are presented as means ± SD (*n* = 12). *P*-values from two-way ANOVA (*p* ≤ 0.05). If interaction was significant, Tukey *post hoc* test was used to identify differences in the experimental treatments. Different capital letters indicate differences among pH regardless time or among times regardless pH.*

### Plasma Humoral Responses

No differences were observed in plasma lysozyme and peroxidase activities (Table 7). Higher concentrations of plasma total proteins were observed after 4 weeks relative to 4 and 24 h, whereas total immunoglobulin levels increased at 4 h relative to 24 h, regardless pH. Senegalese sole exposed to pH 7.3 showed higher antiproteases activity at 24 h relative to the remaining treatments. At 4 weeks, fish reared at pH 8.1 and 7.9 showed increased levels of plasma antiprotease activity relative to those sampled at 4 h, while fish held at pH 7.6 showed higher activity at 4 weeks relative to those sampled at 4 and 24 h. Plasma bactericidal activity decreased at 4 h in fish reared at pH 8.1 relative to those held at pH 7.3 while the opposite pattern was observed at 24 h. At pH 8.1, bactericidal activity was higher at 24 h and 4 weeks compared to 4 h, whereas fish under pH 7.9 and 7.6 presented higher bactericidal activities at 4 weeks relative to 4 h. Lastly, the bactericidal activity of fish from pH 7.3 treatment increased from 24 h to 4 weeks.

**TABLE 7:** Plasma lysozyme (μg mg protein), peroxidase (units ml^–1^), total protein (mg ml^–1^), total immunoglobulins (mg ml^–1^), antiprotease (%), proteases (%) and bactericidal activities (%) of Senegalese sole submitted to different water pH during 4, 24 h and 4 weeks.

Plasma Parameters	pH											
	
	8.1			7.9			7.6			7.3		
	
	4 h	24 h	4 weeks	4 h	24 h	4 weeks	4 h	24 h	4 weeks	4 h	24 h	4 weeks
Lysozyme	1.86 ± 0.39	2.37 ± 0.58	1.09 ± 0.73	2.41 ± 0.24	2.25 ± 0.76	1.75 ± 0.60	2.38 ± 0.51	2.13 ± 0.75	1.77 ± 0.72	2.13 ± 0.63	1.82 ± 0.59	1.27 ± 1.15
Peroxidase	25.19 ± 2.78	38.53 ± 22.48	35.15 ± 21.62	29.02 ± 7.03	51.37 ± 22.42	49.64 ± 25.51	28.63 ± 2.44	124.56 ± 60.61	117.59 ± 63.43	31.07 ± 13.97	33.26 ± 9.32	34.75 ± 9.40
Total Protein	33.51 ± 3.06	30.88 ± 4.75	35.68 ± 2.22	31.49 ± 4.58	33.92 ± 3.60	35.84 ± 3.38	34.75 ± 3.12	32.34 ± 3.45	36.18 ± 4.15	31.00 ± 6.41	33.60 ± 2.18	34.72 ± 3.84
Total Immunoglobulin	17.38 ± 2.12	12.70 ± 6.69	12.72 ± 1.93	14.68 ± 3.57	10.50 ± 4.19	12.68 ± 2.81	18.65 ± 2.82	11.27 ± 4.35	15.06 ± 3.05	13.61 ± 7.68	11.11 ± 1.07	13.41 ± 2.05
Antiprotease activity	71.07 ± 0.32*^§^	67.04 ± 2.13^b^*	77.93 ± 0.81^§^	68.56 ± 1.62*^§^	65.79 ± 2.96^b^*	76.66 ± 1.68^§^	65.81 ± 4.43*	64.11 ± 2.48^b*^	77.16 ± 1.02^§^	67.04 ± 3.46*	76.25 ± 3.92^a^§	70.39 ± 9.80*^§^
Proteases activity	10.65 ± 0.59	10.67 ± 1.87	9.59 ± 0.74	10.08 ± 0.71	10.48 ± 0.71	9.74 ± 0.73	9.71 ± 0.39	9,49 ± 1.09	9.54 ± 1.00	10.29 ± 0.81	10.05 ± 0.73	9.19 ± 0.77
Bactericidal activity	31.14 ± 3.10^b^*	47.63 ±^a^§	44.74 ± 3.98^§^	34.63 ± 5.61^ab^*	43.34 ±7.11^ab^*§	46.93 ± 6.02^§^	40.87 ± 4.06^ab^*	41.13 ± 6.77^ab^*§	52.35 ± 2.73^§^	42.93 ± 4.85^a^*§	34.71 ± 5.02^b^*	50.76 ± 6.71^§^

**Two-way ANOVA**									**Time**			
	
**Parameters**		**pH**		**Time**		**pH × Time**		**4 h**		**24 h**		**4 weeks**

Lysozyme		ns		ns		ns		–		–		–
Peroxidase		ns		ns		ns		–		–		–
Total Protein		ns		0.032		ns		B		B		A
Total Immunoglobulin		ns		0.003		ns		A		B		AB
Antiprotease activity		ns		<0.001		<0.001		B		B		A
Proteases activity		ns		ns		ns		–		–		–
Bactericidal activity		ns		<0.001		<0.001		C		B		A

*Values are presented as means ± SD (*n* = 12). *P*-values from two-way ANOVA (*p* ≤ 0.05). If interaction was significant, Tukey *post hoc* test was used to identify differences in the experimental treatments. Different lowercase letters stand for significant differences among pH treatments for the same time, while different symbols stands for significant differences between times for the same pH. Different capital letters indicate differences among pH regardless time or among times regardless pH.*

### Mucus Humoral Responses

Mucus peroxidase activity decreased at 24 h relative to 4 h while the bactericidal activity increased at 24 h and 4 weeks relative to 4 h in fish exposed to pH 7.9 and at 4 weeks relative to 4 h in those reared at pH 7.6. Fish exposed to pH 8.1 and sampled at 24 h presented higher anti-protease activity compared to the remaining pH treatments and showed lower activity at 4 weeks than at 4 and 24 h (Table 8).

**TABLE 8:** Mucus peroxidase (units protein), total protein (mg ml^–1^), antiprotease (%), proteases (%) and bactericidal activities (%) of Senegalese sole submitted to different water pH during 4, 24 h and 4 weeks.

Mucus Parameters	pH											
	
	8.1			7.9			7.6			7.3		
				
	4 h	24 h	4 weeks	4 h	24 h	4 weeks	4 h	24 h	4 weeks	4 h	24 h	4 weeks
Peroxidase	1.29 ± 0.31	1.14 ± 0.15	1.02 ± 0.13	1.57 ± 0.23	0.88 ± 0.06	1.22 ± 0.19	1.40 ± 0.57	1.17 ± 0.16	1.26 ± 0.10	1.05 ± 0.38	0.96 ± 0.33	1.06 ± 0.10
Total Protein	1.32 ± 0.41	1.28 ± 0.27	1.61 ± 0.34	0.89 ± 0.11	1.71 ± 0.15	1.19 ± 0.09	1.70 ± 1.29	1.41 ± 0.48	1.10 ± 0.09	1.55 ± 0.64	2.03 ± 1.43	1.35 ± 0.16
Antiproteases	62.53 ± 3.67*	71.28 ± 1.04^a^*	57.98 ± 2.34^§^	69.53 ± 4.18	57.93 ± 2.43^b^	62.63 ± 3.30	64.88 ± 7.95	58.01 ± 3.41^b^	62.82 ± 4,24	61.75 ± 9.98	58.32 ± 10.02^b^	62.14 ± 5.57
Proteases	6.52 ± 1.05	6.43 ± 1.72	7.28 ± 1.18	5.38 ± 0.42	7.66 ± 1.29	6.02 ± 1.19	7.57 ± 3.31	7.15 ± 2.05	8.02 ± 0.25	6.99 ± 1.87	9.30 ± 4.50	7.54 ± 2.15
Bactericidal activity	25.41 ± 5.90	44.05 ± 17.23	25.52 ± 9.28	10.43 ± 6.72*	35.50 ± 4.60^§^	45.14 ± 23.90^§^	19.45 ± 7.87*	28.37 ± 6.16*^§^	52.52 ± 16.93^§^	27.63 ± 4.18	31.11 ± 3.18	34.64 ± 7.50

**Two-way ANOVA**									**Time**			
	
**Parameters**		**pH**		**Time**		**pH × Time**		**4 h**		**24 h**		**4 weeks**

Peroxidase		ns		0.005		ns		A		B		AB
Total Protein		ns		ns		ns		–		–		–
Antiprotease activity		ns		ns		0.001		–		–		–
Proteases activity		ns		ns		ns		–		–		–
Bactericidal activity		ns		<0.001		0.002		B		B		A

*Values are presented as means ± SD (*n* = 12). *P*-values from two-way ANOVA (*p* ≤ 0.05). If interaction was significant, Tukey *post hoc* test was used to identify differences in the experimental treatments. Different lowercase letters stand for significant differences among pH treatments for the same time, while different symbols stands for significant differences between times for the same pH. Different capital letters indicate differences among pH regardless time or among times regardless pH.*

### Gene Expression

**FIGURE 1:**
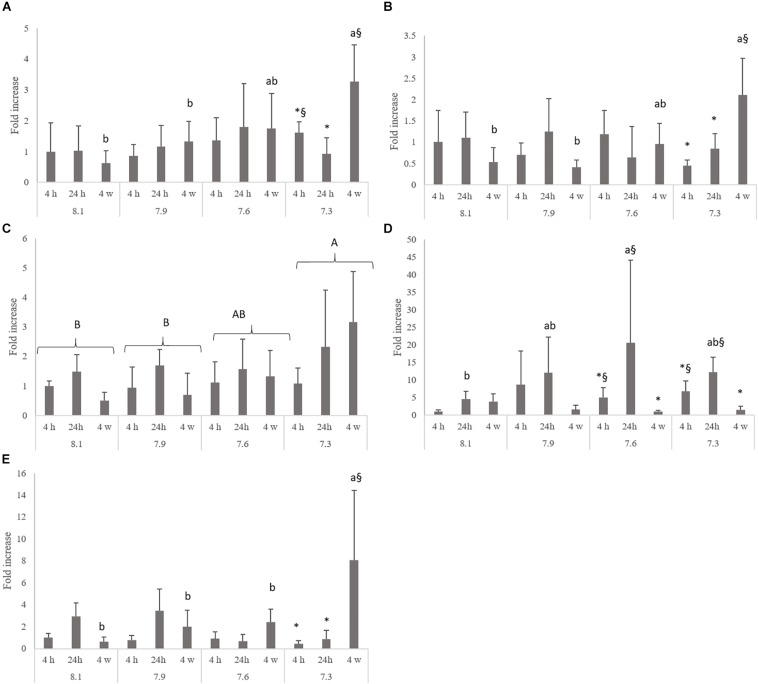
Quantitative expression of interleukin-1β (**A**; *p-value* = 0.032), ciclooxigenase-2 (**B**; *p-value* < 0.001), glucocorticoids receptor 1 (**C**; *p-value* = 0.007), hypoxia inducible factor-1 alpha (**D**; *p-value* = 0.0047) and interleukin 10 (**E**; *p-value* < 0.001) in the head-kidney of Senegalese sole juveniles submitted to pH levels of 8.1, 7.9, 7.6, and 7.3 for 4 and 24 h and 4 weeks. Data are expressed as means ± SD (*n* = 6). Bars represent the fold increase in expression as compared to fish exposure to normocapnia (8.1 pH) at 4 h, previously normalized to endogenous 18S ribosomal (18 s). Different lowercase letters stand for significant differences among pH treatments for the same time, while different symbols stands for significant differences between times for the same pH. Different capital letters indicate differences among pH regardless time or among times regardless pH. (Two-way ANOVA; Tukey *post hoc* test; *p* ≤ 0.05).

The mRNA expression levels of *il1*β (Figure 1A) and *cox2* (Figure 1B) increased in fish exposed to pH 7.3 compared to those exposed to pH 8.1 and 7.9 after 4 weeks. The lowest pH treatment (pH 7.3) also up-regulated *gr1* compared to pH 8.1 and 7.9, but this effect was present regardless of sampling time (Figure 1C). Also, at 4 weeks, mRNA expression levels of *il10* (Figure 1E) augmented in fish exposed to the lowest pH level (pH 7.3) relative to the remaining pH treatments. After 24 h, *hif1* transcripts were higher in fish under pH 7.6 relative to those exposed to pH 8.1 (Figure 1D). Fish held at the lowest pH (7.3) showed higher expression levels of *cox-2* and *il10* at 4 weeks compared to those sampled at 4 and 24 h. The expression of *il1*β was also higher in the same fish group (7.3) but only compared to those sampled at 24 h. Finally, the mRNA expression of *hif1* was enhanced in fish sampled at 24 h compared to those sampled at 4 weeks and held at both pH 7.6 and 7.3.

### Gills Immunofluorescence

**FIGURE 2:**
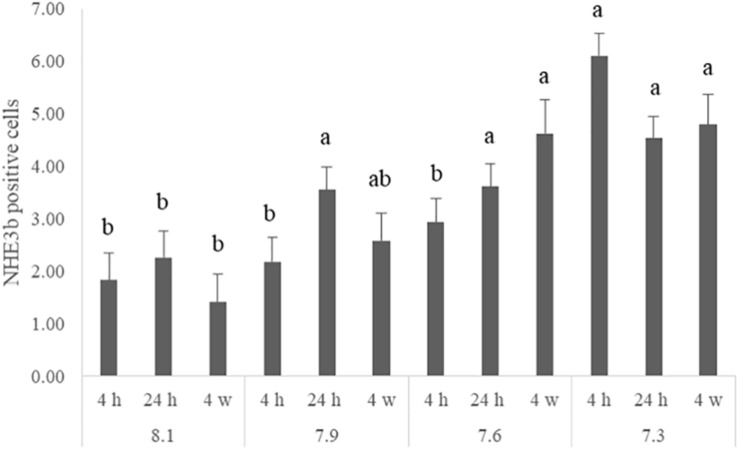
Number of NHE3b positive cells for each quantified filament in the gills of Senegalese sole juveniles submitted to pH levels of 8.1, 7.9, 7.6, and 7.3 for 4 and 24 h and 4 weeks. Data are expressed as means ± SD (*n* = 6; *p-value* = 0.029). Different lowercase letters stand for significant differences among pH treatments for the same time. (Two-way ANOVA; Tukey *post hoc* test; *p* ≤ 0.05).

**FIGURE 3:**
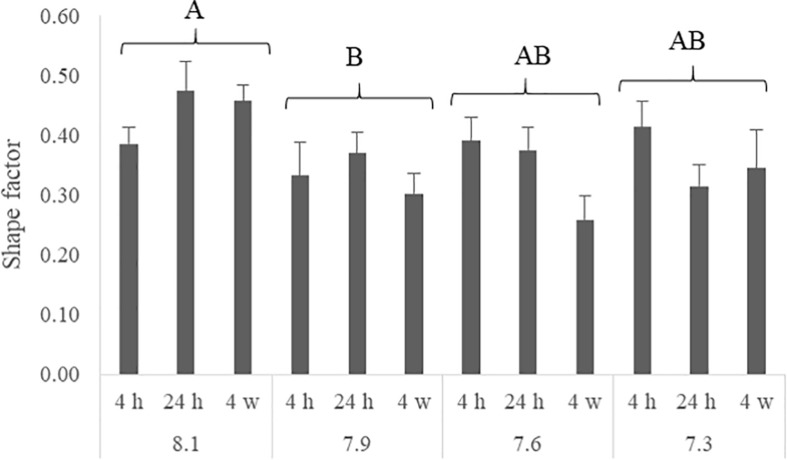
Shape factor of each α5 positive cells in the gills of Senegalese sole juveniles submitted to pH levels of 8.1, 7.9, 7.6, and 7.3 for 4 and 24 h and 4 weeks. Data are expressed as means ± SD (n = 6; *p-value* = 0.001). Different capital letters indicate differences among pH regardless time or among times regardless pH. (Two-way ANOVA; Tukey *post hoc* test; *p* ≤ 0.05).

**FIGURE 4:**
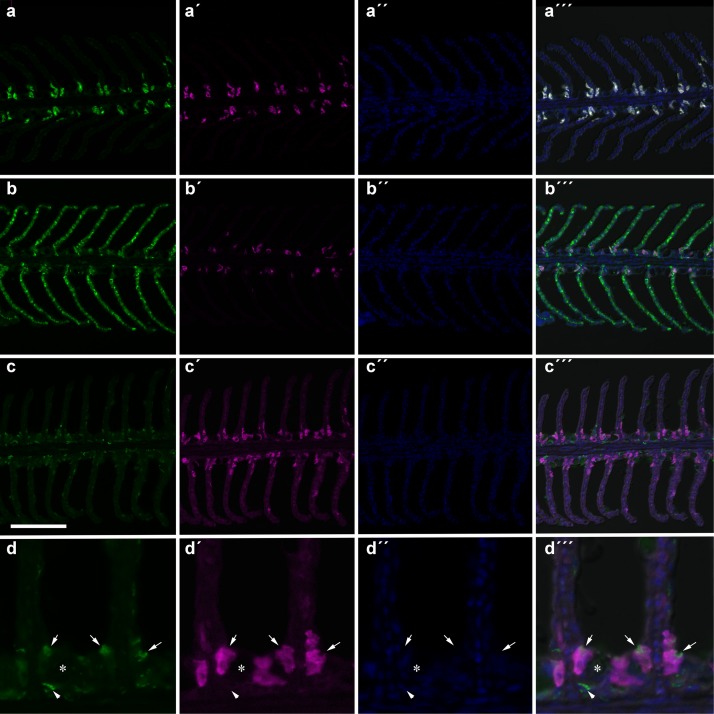
Immunohistochemical colocalization of NKA **(a–d)** with NKCC **(a′–a″′)**, CA **(b′–b″′)** and NHE3b **(c′–c″′,d′–d″′)** in the gills of Senegalese sole. All sections are counter stained with DAPI **(a″,b″,c″,d″**) and merged with the DIC image **(a″′,b″′,c″′,d″′)**. A higher (4x) magnification of the NHE3b-NKA staining is shown in **(d–d″′)**. Arrows indicate apical NHE3b of NKA-immunoreactive (IR) cells. Arrows head indicate a NHE3b-IR cell deep within the filament epithelium. The asterisk indicates a goblet cell. Scare bar 100 μm **(a–c)**; 25 μm **(d)**.

The number of NHE3b positive gill ionocytes (NKA-immunoreactive cells) per gill filament section increased with lowest water pH (Figure 2). At 4 h of exposure, a higher amount of NHE3b positive cells was found in fish exposed to pH 7.3 relative to the remaining pH treatments, while after 24 h all the CO_2_ treatment groups (pH 7.3, 7.6 and 7.9) presented higher amounts of NHE3b positive cells compared to those observed with pH 8.1 control group. Moreover, after 4 weeks the number of NHE3b positive cells in fish exposed to both pH 7.3 and 7.6 continued enhanced relative to those observed in fish held at pH 8.1. Data for the shape factor of the whole cell (NKA immunoreactivity) (Figure 3) characterizes the degree of deviation from an ideal shape (a circle shape) normalized to a range of zero to one where one is closest to the ideal shape. The only difference observed was a lower shape factor in fish exposed to pH 7.9 compared to the pH 8.1 control group. No interactions were observed. There was no noticeable difference in area or intensity of immunofluorescence or in the number of immunoreactive cells (ionocytes) among the treatment for any other antibody tested. Example of gills immunohistochemical colocalization of NKA with NKCC, CA and NHE3b in the gills of Senegalese sole presented in Figure 4.

### Respirometry

No differences were found in SMR or in MMR values of any experimental group whereas fish submitted to pH 7.3 presented a reduced AMS compared to those maintained at pH 8.1 (i.e., the control group) (Table 9). These findings indicated reduced aerobic performance among the fish exposed to the lowest pH.

**TABLE 9:** Standard metabolic rate (SMR), maximum metabolic rate (MMR), and aerobic metabolic scope (AMS) (mg O_2_ kg^–1^ h^–1^) in Senegalese sole submitted to different water pH (8.1, 7.9, 7.6, and 7.3).

Parameters	pH				One-way ANOVA (*p-value*)
		
	8.1	7.9	7.6	7.3	
SMR	60.24 ± 10.18	73.27 ± 17.95	77.09 ± 10.63	55.58 ± 12.19	ns
MMR	119.47 ± 14.55	120.95 ± 19.68	122.53 ± 16.03	94.51 ± 9.59	ns
AMS	59.231 ± 4.97^a^	47.68 ± 7.56^ab^	45.44 ± 11.86^ab^	38.93 ± 11.02^b^	0.050

*Values are presented as means ± SD (*n* = 6). *P*-values from one-way ANOVA (*p* ≤ 0.05). If interaction was significant, Tukey *post hoc* test was used to identify differences in the experimental treatments. Different lowercase letters stand for significant differences among pH.*

## Discussion

Increasing water CO_2_ in a RAS scenario or due to anthropogenic release leads to physiological adaptations in fish activating regulatory mechanism toward acidosis compensation. Findings of the present study pointed to a clear impact on immune mechanisms, gills and aerobic performance, mainly after chronic exposure to extreme hypercapnic water experienced in fish farming, with the outcome of negatively impacting Senegalese sole growth.

Acid-base regulation allows the well- functioning of basic metabolic mechanisms when homeostasis is challenged by environmental changes in the respiratory gases, carbon dioxide and oxygen. Small modifications of hematological and innate-immune mechanisms could allow fish to respond to this external stressor and ultimately restoring balance. Since the effects of short-term hypercapnic exposure are expected to be mitigated by slight physiological modifications (Cech and Crocker, 2002; Heuer and Grosell, 2014; Esbaugh, 2018), and the disturbance rapidly alleviated, few works have targeted this subject from an immunological perspective, assessing the direct effect on the immune mechanisms. The present study supports in fact this observation, since no differences were observed regarding hematological parameters nor peripheral leukocyte population after only 4 and 24 h of exposure to low water pH. Nonetheless, the increased plasma total bactericidal and antiproteases activities observed at 4 and 24 h of exposure, respectively, in Senegalese sole exposed to the lowest pH treatment (7.3) possibly reflected an immune response adjustment to the water acidification-induced stress. Also, skin mucus antiprotease activity decreased at 24 h in all hypercapnia treatments thus showing an effect of acute hypercapnia exposure also on the external mucosal barrier. Mucosal surfaces (i.e., skin, gills and gut) provide fish the first line of defense, and skin mucus, similarly to peripheral blood, is armed with potent molecules, including lysozymes, complement proteins, lectins and antimicrobial peptides with important immune roles (Cabillon and Lazado, 2019). In this study, the modulation of humoral parameters found both in skin mucus and plasma seem to suggest some degree of adjustment to the acidic ambient water, even after a short period.

By being in direct contact with the external environment, CO_2_ is rapidly sensed by chemoreceptors in fish gills, which then trigger compensatory mechanisms toward the acidosis by transporting acid and base equivalents in and out the body through branchial ionocytes (Heuer and Grosell, 2014). In fact, extreme hypercapnia for short-periods can severely disrupt acid-base balance and gas transfer across fish gills in the same way as a long-term exposure to intermediate hypercapnia levels (Randall and Daxboeck, 1984). In the present work, the gills of fish exposed to the lowest water pH (7.3) for only 4 h displayed an increased number of Na^+^/H^+^ exchanger (NHE3b) positive ionocytes which are likely involved in acid secretion as a physiological adaptation acidosis compensation (Evans et al., 2005). Though these cells progressively decreased with time at pH 7.3, their value is nonetheless higher than the control treatment group. Moreover, fish reared in the remaining hypercapnia treatments (pH 7.9 and 7.6) presented even higher frequencies of NHE3b positive cells than the control at 24 h. NHE3 is involved in the system that mediates transepithelial pH regulation which makes uses of the inward Na^+^ gradient to drive the excretion of H^+^ in ionocytes (Claiborne et al., 2002). Seidelin et al. (2001) reported that its mRNA expression and protein abundance is consistent with a need for acid excretion upon exposure to hypercapnia. However, at the lower levels (pH 7.9) of hypercapnia typical of ocean acidification conditions, acidosis can be compensated by increased HCO_3_^–^ retention without an increase in NHE expression (Heuer and Grosell, 2014).

In the present study, the gene coding for the hypoxia inducible factor-1α (*hif1*) was found up- regulated at 24 h in the head-kidney of fish held at pH 7.6 relative to the control while being maintained for the remaining levels of hypercapnia. HIF is the prime regulator of the transcriptional response to oxygen availability and is also responsible for regulating inflammatory genes transcription (Schofield and Ratcliffe, 2004). As the product of oxidative metabolism, CO_2_ shares an inverse relationship with O_2_ levels. Moreover, in a normoxic situation HIF-1 is constitutively marked for degradation by prolyl hydrolases (Semenza, 1999), which in turn targets HIF-1α subunit for ubiquitination and degradation (Schofield and Ratcliffe, 2004). In contrast, under hypoxic conditions and when CO_2_ is higher, HIF-1α degradation is diminished (Selfridge et al., 2016). In the present study, water oxygen levels were stable in all hypercapnic treatments, which could explain the general lack of clear results found in HIF-1α mRNA expression since this mechanism is controlled by oxygen. Furthermore, Costas et al. (2011) observed that HIF-1α expression could be augmented in response to a stress, induced by repeated handling, which could elucidate the observed *hif1* up-regulation in fish exposed to pH 7.6. HIF-1 is described by Cramer et al. (2003) as essential for the maintaining the ATP pool and proper functioning of myeloid cells activity, as neutrophils and monocytes. Therefore, the slight increase of *hif1* expression in the hypercapnic treatments (only significantly higher at pH 7.6 at 24 h) together with higher plasma bactericidal and anti-proteases activities could be a result of an acute stress recognition and response.

In response to the prolonged exposures (4 weeks), all hypercapnic treatments displayed a higher plasma antiproteases activity relative to the control group. Additionally, mRNA levels of the pro-inflammatory cytokine IL1β and cyclooxygenase 2, COX2, an enzyme involved in the production of prostaglandins in response to inflammation, were found up- regulated in the highest hypercapnic treatment (pH 7.3) relative to pH 8.1 and to those exposed to the lowest hypercapnic treatment, pH 7.9. Also, gene expression levels of the anti-inflammatory cytokine IL10 increased at pH 7.3 compared to all other treatments. In contrast to that observed at 24 h in response to water acidification, the expression of HIF-1α decreased in fish exposed to the two lowest pH treatments. These observations revealed a marked effect of water CO_2_ increase in the fish immune mechanisms with the up-regulation of important pro- and anti-inflammatory mediators and stress-related genes. In fact, gill NHE3b positive ionocyte numbers remained high under hypercapnic treatments even after a 4 weeks period. It is therefore suggested that, in response to severe hypercapnia, the physiological compensatory mechanisms are activated toward a chronic stress response, possibly resulting in the diverting of energy away from growth. In fact, a clear reduction of sole final body mass was observed in response to the decrease of water pH, particularly at pH 7.3. This is supported by Foss et al. (2002) and Mota et al. (2019) who showed a reduced growth in juvenile spotted wolfish (*Anarhichas minor* Olafsen) and Atlantic salmon, respectively, with an increase of water CO_2_. Moreover, Cech and Crocker (2002) found impaired growth of white sturgeon (*Acipenser transmontanus*) reared at pH 7.0 compared to normocapnia (pH 8.0).

Finally, SMR and MMR provide information regarding lower and upper limits for aerobic energy metabolism. Despite the lack of clear effects of the increase water CO_2_ on the standard and MMRs, fish AMS was found to be diminished in the lowest pH treatment (pH 7.3). The progressive decrease of AMS shows a gradual reduction of the aerobic performance available for the normal metabolism in extreme hypercapnic situations, possibly as a result of the expenditure of metabolic energy toward the immune system combined with decreasing trends in the MMR as the pH declined. Supporting this hypothesis, and as previously demonstrated by Claireaux et al. (2000) for Atlantic cod (*Gadus morha*), a positive correlation between final growth and aerobic metabolic scope (*r*^2^ = 0.896) was found. Moreover, the observed reduction of the aerobic performance (i.e., AMS) by the low pH exposure could have reduced the energy availability for meal digestion and assimilation, or the specific dynamic action (SDA) as has been measured in eels (Methling et al., 2013). This could also have contributed to the reduced growth observed in the low pH treatments.

The present results showed a marked effect of increased water CO_2_ on Senegalese sole with changes in gills physiology, immune mechanisms and aerobic performance. The increase of humoral immune parameters and up-regulation of important inflammatory mediators and stress-related genes after both acute and prolonged exposure to low pH pointed to a clear physiological adaptation of fish toward the acidic water. This physiological adaptation together with lower AMS and growth impairment could be the result of energy deviation toward the immune response to water hypercapnia.

## Data Availability Statement

The datasets generated for this study are available on request to the corresponding author.

## Ethics Statement

The animal study was reviewed and approved by this study was directed by trained scientists (following FELASA category C recommendations) and conducted according to the guidelines on the protection of animals used for scientific purposes from the European directive 2010/63/UE.

## Author Contributions

MM, FA, JS, and BC conceived the experiments. MM and FA conducted the experimental trial. LP and JW performed the gill immunohistochemistry. MM, RA, and JS performed the respirometry work. RA assisted with analytical procedures. MM directed most laboratory techniques and wrote the manuscript under the supervision of BC. All authors contributed to and approved the manuscript.

## Conflict of Interest

The authors declare that the research was conducted in the absence of any commercial or financial relationships that could be construed as a potential conflict of interest. The handling Editor declared a past collaboration with one of the authors BC.
